# Genome-wide association mapping including phenotypes from relatives without genotypes in a single-step (ssGWAS) for 6-week body weight in broiler chickens

**DOI:** 10.3389/fgene.2014.00134

**Published:** 2014-05-20

**Authors:** Huiyu Wang, Ignacy Misztal, Ignacio Aguilar, Andres Legarra, Rohan L. Fernando, Zulma Vitezica, Ron Okimoto, Terry Wing, Rachel Hawken, William M. Muir

**Affiliations:** ^1^Genus plcHendersonville, TN, USA; ^2^Department of Animal and Dairy Science, University of GeorgiaAthens, GA, USA; ^3^Instituto Nacional de Investigación Agropecuaria, INIA Las Brujas, Mejoramiento Genético AnimalCanelones, Uruguay; ^4^Institut National de la Recherche Agronomique, UR631 Station d'Amélioration Génétique des AnimauxCastanet-Tolosan, France; ^5^Department of Animal Science, Iowa State UniversityAmes, IA, USA; ^6^Département de Sciences Animales, Ecole Nationale Superieure Agronomique de Toulouse, Université de ToulouseCastanet-Tolosan, France; ^7^Cobb-Vantress Inc.Siloam Springs, AR, USA; ^8^Department of Animal Science, Purdue UniversityWest Lafayette, IN, USA

**Keywords:** body weight, broiler chicken, genome-wide association, ssGWAS, BayesB, association mapping

## Abstract

The purpose of this study was to compare results obtained from various methodologies for genome-wide association studies, when applied to real data, in terms of number and commonality of regions identified and their genetic variance explained, computational speed, and possible pitfalls in interpretations of results. Methodologies include: two iteratively reweighted single-step genomic BLUP procedures (ssGWAS1 and ssGWAS2), a single-marker model (CGWAS), and BayesB. The ssGWAS methods utilize genomic breeding values (GEBVs) based on combined pedigree, genomic and phenotypic information, while CGWAS and BayesB only utilize phenotypes from genotyped animals or pseudo-phenotypes. In this study, ssGWAS was performed by converting GEBVs to SNP marker effects. Unequal variances for markers were incorporated for calculating weights into a new genomic relationship matrix. SNP weights were refined iteratively. The data was body weight at 6 weeks on 274,776 broiler chickens, of which 4553 were genotyped using a 60 k SNP chip. Comparison of genomic regions was based on genetic variances explained by local SNP regions (20 SNPs). After 3 iterations, the noise was greatly reduced for ssGWAS1 and results are similar to that of CGWAS, with 4 out of the top 10 regions in common. In contrast, for BayesB, the plot was dominated by a single region explaining 23.1% of the genetic variance. This same region was found by ssGWAS1 with the same rank, but the amount of genetic variation attributed to the region was only 3%. These findings emphasize the need for caution when comparing and interpreting results from various methods, and highlight that detected associations, and strength of association, strongly depends on methodologies and details of implementations. BayesB appears to overly shrink regions to zero, while overestimating the amount of genetic variation attributed to the remaining SNP effects. The real world is most likely a compromise between methods and remains to be determined.

## Introduction

Genome-wide association analysis (GWAS) is an efficient way to discover QTLs associated with phenotypes. A common method for GWAS is to sequentially fit all SNPs one at a time as a fixed effect in a mixed model that includes the pedigree or genomic relationship matrix to control for polygenic background effects (Meyer and Tier, 2012; Xie et al., 2012). Another method utilizes all markers simultaneously using a Bayesian framework (Abasht et al., 2009). If the phenotypes used in these analyses are BLUP estimates of breeding values, both methods may have severe limitations. When much of the phenotypic information is on ungenotyped animals, the BLUP estimates need to be de-regressed (DP) (Garrick et al., 2009), which can lead to biases and losses of accuracy (Vitezica et al., 2011; Ricard et al., 2013). Calculation of DP relies on accuracies of estimated breeding values. For large data sets, such accuracies cannot be computed directly and need to be approximated and for complex models and data structures, such approximations may be poor or unavailable (Sanchez et al., 2008).

An alternative GWAS approach was recently proposed by Wang et al. (2012) where all genotypes, observed phenotypes and pedigree information are jointly considered in one step (ssGBLUP), and thus allows the use of any model, and all relationships simultaneously. With this approach, all SNPs are considered simultaneously along with all phenotypes from those genotyped and ungenotyped. The latter is accomplished by augmenting the genomic relationships with traditional pedigree relationships (Aguilar et al., 2010). With this approach GWAS is accomplished by converting the estimated breeding values (GEBVs) obtained from ssGBLUP to marker effects and marker weights, which are then used in an iterative approach to update solutions. The theoretical advantage of this method is that it uses all phenotypic information for which either the pedigree or marker effects are known, and can be used for any model for which BLUP estimates of breeding values can be obtained (Wang et al., 2012). GWAS by ssGBLUP can be called ssGWAS. Dikmen et al. (2013) applied ssGWAS for identification of QTLs for rectal temperature during heat stress in Holsteins using a model with many effects.

Wang et al. (2012) examined the efficiency of the ssGWAS method by simulation. In those simulations, ssGWAS achieved the highest correlation between QTL effect and the sum of 8 adjacent SNP effects as compared to BayesB (Habier et al., 2011) or classical GWAS (Meyer and Tier, 2012), while at the same time was faster and simpler to apply than other methods. However, simulated data may not reflect true world genetic architectures, such as LD patterns, allelic effect distributions, and number of loci affecting the trait, which may affect the comparisons. The objective of this study was to compare the same three GWAS methods using real data to detect QTLs for body weight at 6 weeks (BW6) in broiler chickens.

## Materials and methods

Animal Care and Use Committee approval was not obtained for this study because the data were obtained from an existing database.

### Data

Body weights at 6 weeks (BW6) for broiler chickens were provided by Cobb-Vantress Inc. (Siloam Springs, AR) for a dam line across 5 generations (G1, G2 G3, G4, and G5). The total number of animals with phenotypes was 274,776, and the average BW6 was 2.40 ± 0.33 kg. Complete pedigrees were available for all individuals. For generations G1–G4, 4732 broilers were genotyped with 57,636 SNP markers on a SNP panel across the whole genome developed by Groenen et al. (2011).

Quality control (QC) procedures were applied to remove genotyped individuals with pedigree errors and SNP genotypes that were either monomorphic, or displaced segregation distortion according to Wiggans et al. (2010) with methodology by Aguilar et al. (2011). After QC, 179 birds were removed for pedigree errors and 4553 birds (2205 in G1, 737 in G2, 818 in G3, 793 in G4) with 40,615 autosomal SNPs remained in the data set. Moreover, the number of SNP loci with missing genotypes reduced from 29.8 to 0.32%.

### Models and computation

#### Single-step genomic association study

The ssGWAS method is a modification of BLUP with the numerator relationship matrix **A**^−1^ matrix replaced by **H**^−1^ (Aguilar et al., 2010):

$$H^{-1}=A^{-1}+\left[\begin{array}{cc} 0 & 0\\ 0 & G^{-1}-A_{22}^{-1} \end{array}\right]$$

where *A*_22_ is a numerator relationship matrix for genotyped animals and *G* is a genomic relationship matrix. The genomic matrix can be created following Vanraden (2008) as:

G = ZDZ'q

where *Z* is a matrix of gene content adjusted for allele frequencies, *D* is a weight matrix for SNP (initially *D* = *I*), and q is a weighting factor. The weighting factor can be derived either based on SNP frequencies (Vanraden, 2008), or by ensuring that the average diagonal in G is close to that of *A*_22_ (Vitezica et al., 2011). The latter method was used in this study. Briefly, SNP effects and weights for GWAS were be derived as follows (Wang et al., 2012):

1. Let D = I in the first step.
2. Calculate G = ZDZ′q.
3. Calculate GEBVs for entire data set using ssGBLUP.
4. Convert GEBVs to SNP effects (*û*): *û* = *qDZ*′(*ZDZ*′*q*)^−1^â, where â is the GEBVs of animals which were also genotyped.
5. Calculate weight for each SNP: *d*_*i*_ = *û*^2^_i_2*p*_*i*_(1−*p*_*i*_), where i is the i-th SNP.
6. Normalize SNP weights to remain the total genetic variance constant.
7. Loop to 2. (ssGWAS1) or 4. (ssGWAS2).

SNP weights were calculated iteratively either looping through steps 4–6 (ssGWAS1) or through steps 2–6 (ssGWAS2). Iterations with both scenarios increase weights of SNP with large effects and decrease those with small effects, essentially regressing them to the mean. Experiences with simulated data using ssGBLUP (Wang et al., 2012) and of a similar method based on GBLUP (Sun et al., 2012; Zhang et al., 2012) indicated that ssGWAS1 was more suitable for identification of SNPs with the largest effects while ssGWAS2 was superior for more accurate GEBVs.

Percentage of genetic variance explained by i-th region has been calculated as below:

$$\frac{Var\left(a_{i}\right)}{\sigma_{a}^{2}}\times 100\%=\frac{Var(\sum_{j=1}^{20}Z_{j}{\hat{u}}_{j})}{\sigma_{a}^{2}}\times 100\%$$

Where *a*_*i*_ is genetic value of the *i*-th region that consists of contiguous 20 SNPs, σ^2^_*a*_ is the total genetic variance, **Z**_*j*_ is vector of gene content of the *j*-th SNP for all individuals, and *û*_*j*_ is marker effect of the *j*-th SNP within the *i*-th region.

#### Computations

For analyses, we applied an animal model with fixed effects of sex and contemporary group and random effects for additive animal and maternal permanent environment. Variance components were estimated by REML based on all the individuals in the pedigree. All analyses for REML, BLUP and ssGWAS were run using the BLUPF90 software (Misztal et al., 2002; Aguilar et al., 2011). For BayesB and CGWAS methods, DP were created from BLUP estimates of EBVs as pseudo-observations, following Garrick et al. (2009) assuming that 0.1 of the genetic variance was not accounted for by SNPs, as in Ostersen et al. (2011). GWAS was then performed using three alternative methods. The first method of ssGWAS was run for five iterations with both ssGWAS1 and ssGWAS2 options. The second method of CGWAS was implemented in WOMBAT (Meyer and Tier, 2012). The third method, BayesB was implemented in GenSel (Habier et al., 2011) with π = 0.9. Estimates of genotypic and residual variances from REML were used as priors in BayesB, which followed a scaled inverse Chi-squared distribution with default parameters used in GenSel. The use of the default parameter π = 0.9 was due to failure for convergence of π estimation based on BayesCπ after 100,000 iterations. A Monte Carlo Markov Chain was completed for 51,000 rounds with Gibbs sampling, of which the first 1000 rounds were discarded as burn-in. Within each Gibbs sample cycle, Metropolis-Hastings samples were run for 10 iterations. Because WOMBAT and GenSel are not able to incorporate missing genotypes, missing SNPs were replaced by their average value for that locus.

Results were compared based on the proportion of total variance explained by the SNP. However, such estimates based on single-SNP were found to be noisy from all the methods due to the high ratio between the number of SNPs and the number of genotyped individuals. Therefore, non-overlapping windows of 20 consecutive SNPs were used to present results in Manhattan plots, instead of single locus. The methods were also compared based on the top 10 ranking windows for genetic variance explained by that window.

The methods were additionally compared in terms of predicted GEBVs. For ssGWAS, GEBVs were obtained directly, whereas for CGWAS and BayesB, GEBVs were calculated as the sum of estimated SNP effects for each genotyped individual. For comparison of accuracy of phenotypic BLUP and ssGWAS, realized accuracies were computed for ssGWAS1 and ssGWAS2, as the ratio of predictive ability over the square root of heritability according to Legarra et al. (2008).

## Results and discussion

### Genetic estimations

Variance components, calculated from regular phenotypic BLUP based on all individuals in the data set, for maternal permanent environmental, additive and residual variances, were respectively 0.20, 1.14, and 3.88. The estimated heritability of BW6 was 0.22, which was similar to earlier estimates using the same trait (Chen et al., 2011).

Table 1 includes correlations between EBV (obtained from regular BLUP) and GEBVs for genotyped individuals; solutions for GEBVs in ssGWAS1 do not change between iterations, and they are the same as the first iteration in ssGWAS2 (ssGWAS2/1). The correlations between EBVand GEBVs for ssGWAS2 in the first and second iterations, and BayesB, were all ≤0.9; while for CGWAS, and ssGWAS with 3 or more iterations, the correlations were <0.9. As SNP effects are calculated in CGWAS individually, estimates for closely linked SNP were similar, which results in correlated residual and is likely to cause problems due to double counting. The decline in correlations after 2 iterations implies the estimates were over-regressing, resulting in lower accuracy.

**Table 1:** **Correlations of EBV obtained from regular BLUP and GEBVs^a^ obtained from three approaches^b^ for genotyped individuals**.

**Correlation**	**EBV**
ssGWAS2/1^c^	0.91
ssGWAS2/2	0.90
ssGWAS2/3	0.88
ssGWAS2/4	0.87
ssGWAS2/5	0.85
BayesB^d^	0.90
CGWAS	0.71

a *GEBVs = genomic breeding values*.

b *Single-step genomic analyses (ssGWAS), BayesB, and classical genome wide association (CGWAS)*.

c *ssGWAS2/1 = the first iteration of Scenario 2 (ssGWAS2) in ssGWAS, which is equivalent to ssGWAS1*.

d *BayesB with π = 0.9*.

Table 2 gives realized accuracies of EBV estimated using phenotypic BLUP and GEBVs estimated using ssGWAS. For these data, the accuracy was maximized by the second iteration (ssGWAS2/2), then declined after the third iteration (ssGWAS2/3). For GBLUP, Sun et al. (2012) added a constant in the equation to calculate SNP variance, mimicking the structure of such formulas in REML. Subsequently, the accuracy reached plateau but did not decline with iterations. In our studies, involving such a constant (results not shown), the accuracy did not improve over ssGWAS2/2 with the original formula. Further, adding a constant makes identification of top QTL more difficult (Sun et al., 2011).

**Table 2:** **Comparison of accuracies of EBV obtained from regular BLUP and GEBVs^a^ from ssGWAS2^b^ with up to 5 iterations**.

**Methods**	**Accuracy**
EBV	0.34
ssGWAS2/1^c^	0.44
ssGWAS2/2	0.52
ssGWAS2/3	0.52
ssGWAS2/4	0.51
ssGWAS2/5	0.50

a *GEBVs = genomic breeding values*.

b *ssGWAS = single-step genomic association analyses*.

c *ssGWAS2/1 = the first iteration of Scenario 1 (ssGWAS1) in ssGBLUP, which is equivalent to ssGWAS2/1*.

### QTL mapping

**Figure 1:**
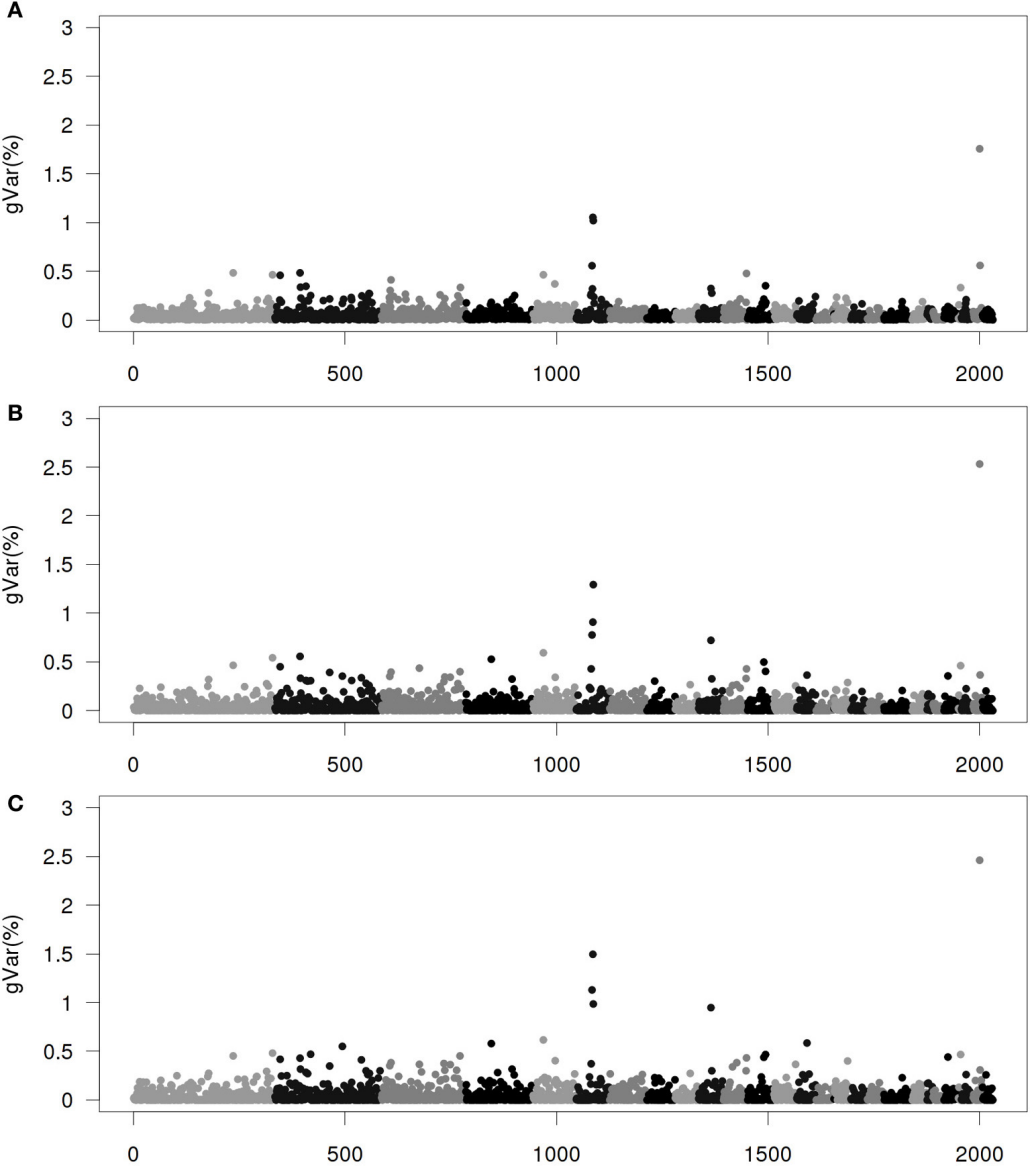
**Proportion of genetic variance of 20-SNP region under the Senarios 1 (ssGWAS1) of extended single-step genomic BLUP (ssGBLUP). (A)** The first iteration (ssGWAS1/1). **(B)** The third iteration (ssGWAS1/3). **(C)** The fifth iteration (ssGWAS1/5). The x-axis represents region location of 20 SNPs. The y-axis represents the proportion of genetic variance of each region.

**Figure 2:**
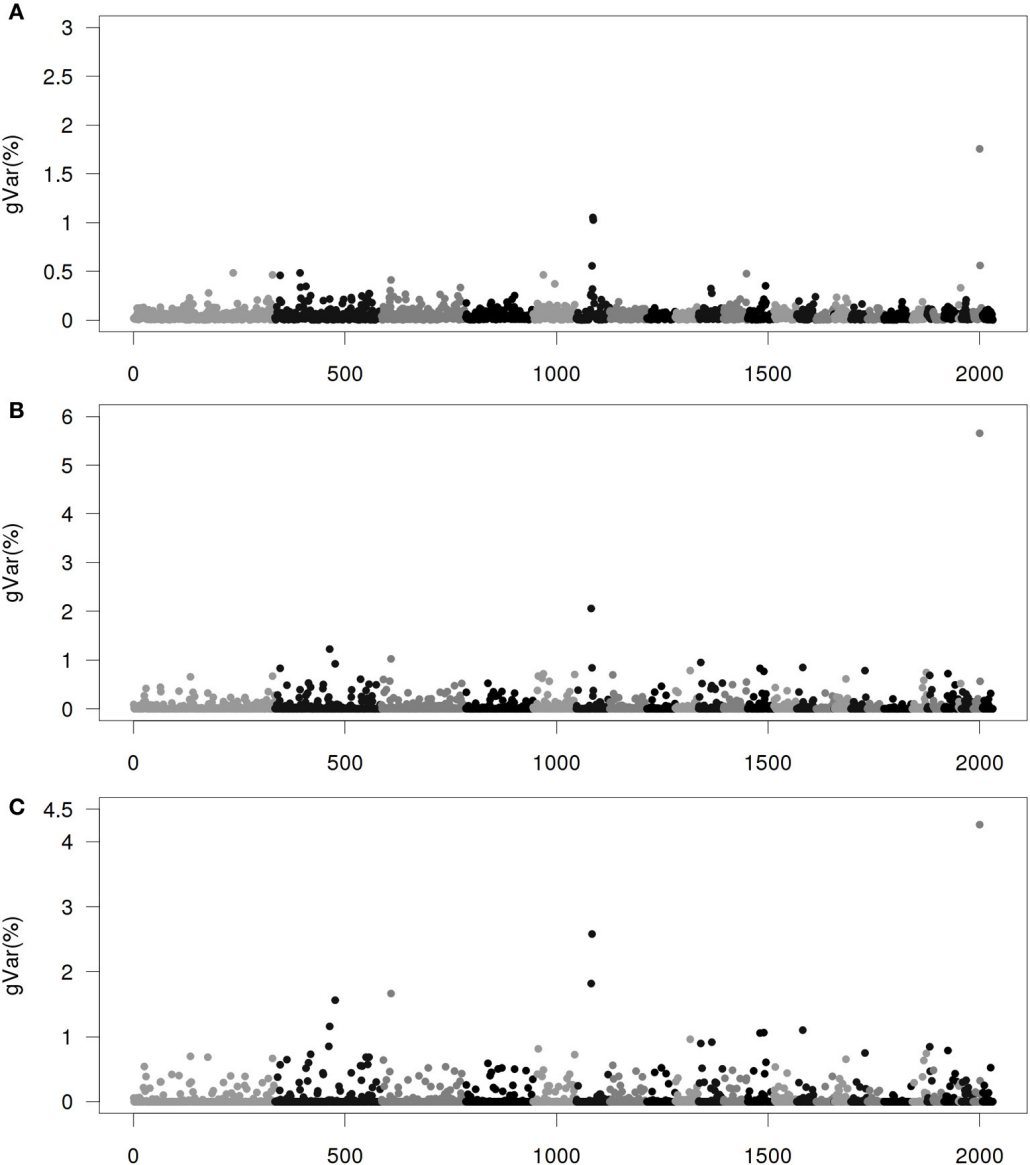
**Proportion of genetic variance of 20-SNP region under the Senarios 2 (ssGWAS2) of extended single-step genomic BLUP (ssGBLUP). (A)** The first iteration (ssGWAS2/1). **(B)** The third iteration (ssGWAS2/3). **(C)** The fifth iteration (ssGWAS2/5). The x-axis represents region location of 20 SNPs. The y-axis represents the proportion of genetic variance of each region.

**Figure 3:**
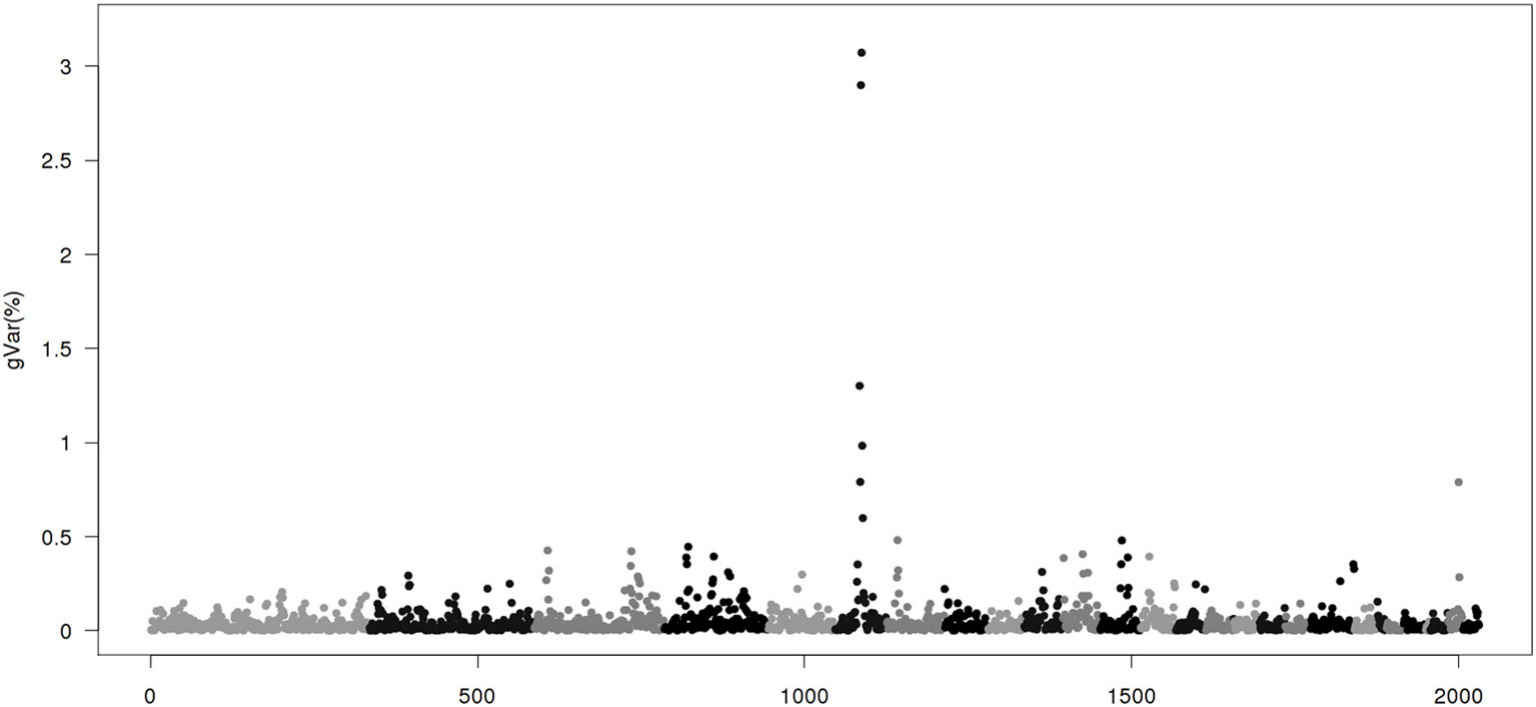
**Proportion of genetic variance of 20-SNP region using classical genome wide association studies (CGWAS) implemented by WOMBAT**. The x-axis represents region location of 20 SNPs. The y-axis represents the proportion of genetic variance of each region.

**Figure 4:**
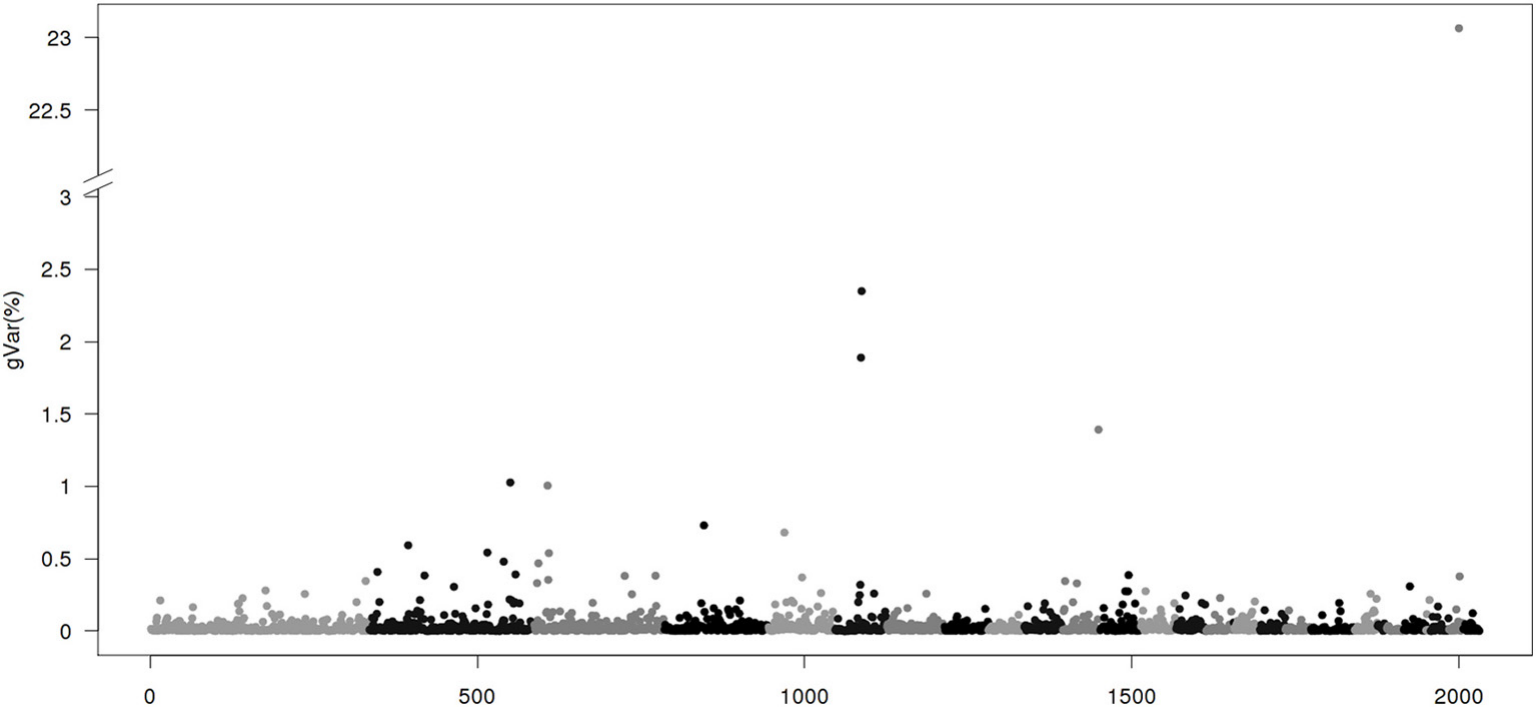
**Proportion of genetic variance of 20-SNP region using BayesB with π = 0.9 implemented by GenSel**. The x-axis represents region location of 20 SNPs. The y-axis represents the proportion of genetic variance of each region.

Figures 1–4 show plots of genetic variances accounted for by windows of 20 contiguous SNPs within a chromosome, based on different methods. Windows were neither overlapping nor repetitive. Chromosomes were differentiated by different shades. In total, there were 2031 regions, with an average chromosomal length of 0.45 Mbp.

Figure 1 shows plots by ssGWAS1/1, ssGWAS1/3, and ssGWAS1/5, which indicate iteration 1, 3, and 5 using ssGWAS1, which derives weights and solely iterates on SNPs. On one hand, as the iterations progress, the plots became less noisy, and the peaks associated with the largest regions become more distinct. On the other hand, the iterations caused some re-rankings of the top regions (Table 3). The simulation studied by Sun et al. (2011) indicated that a few iterations similar to ssGWAS1 provided the most accurate identification of the top QTLs.

**Table 3:** **Rankings of top 10 regions^a^ for 5 iterations in ssGWAS^b^**.

**ssGWAS1/1 (ssGWAS2/1)^c^**	**1**	**2**	**3**	**4**	**5**	**6**	**7**	**8**	**9**	**10**
ssGWAS1/2	1	3	2	12	4	9	7	10	5	6
ssGWAS1/3	1	3	2	21	4	11	7	15	8	6
ssGWAS1/4	1	3	2	32	4	14	10	21	9	6
ssGWAS1/5	1	2	4	36	3	14	19	18	10	6
ssGWAS2/2	1	9	6	2	16	20	19	8	7	5
ssGWAS2/3	1	110	62	29	8	233	57	31	21	16
ssGWAS2/4	1	351	256	72	3	575	126	58	22	35
ssGWAS2/5	1	479	472	100	2	766	179	86	25	50

a *Each region consists of 20 SNPs, and in totally there are 2031 regions on whole genome*.

b *ssGWAS = single-step genomic association analyses*.

c *ssGWAS1/1 = the first iteration of Scenario 1 (ssGWAS1) in ssGBLUP, which is equivalent to ssGWAS2/1*.

Figure 2 shows plots by ssGWAS2/1, ssGWAS2/3, and ssGWAS2/5 iterating on both SNPs and GEBVs. Please note that the plots for ssGWAS1/1 and ssGWAS2/1 are identical. Compared with ssGWAS1, “thinning” in ssGWAS2 is more rapid. The plot of ssGWAS2/3 clearly points to many distinct regions, while the plot in ssGWAS1/3 seems less so. Note that the accuracy of GEBVs peaked at ssGWAS2/2 to ssGWAS2/3 and declined thereafter, suggesting that plots of ssGWAS2/2 and ssGWAS2/3 are also the most accurate depictions of where the most important regions are.

Figure 3 gives results from the CGWAS method. With this method, more peaks were found than from ssGWAS. However, the two largest regions remained the same as ssGWAS1/1-ssGWAS1/5. The presence of many more peaks in CGWAS than the other methods is most likely a result of strongly linked regions resulting in false positive (Shen et al., 2013).

Figure 4 gives results for the BayesB method. The plot is dominated by a few large regions, with all the other regions representing much smaller variances ≤2.5%. Methods like BayesB are strongly influenced by priors (Van Hulzen et al., 2012), and particularly by the percentage of SNPs assumed to have null effect (π). Studies on the number of genes influencing a quantitative effect estimate the number of <500 (Otto and Jones, 2000; Hayes and Goddard, 2001). Here, we assume that 10% of all SNPs (>4000) have effects. However, some of the alleles are rare variants that are not fully captured by medium or even high density SNP panels (Vinkhuyzen et al., 2012). For populations with small effective size, gains in GEBVs over EBVs in genomic selection are partly due to accounting for major genes, and partly for superior genetic relationships among animals. Fitting single chromosome in a genomic evaluation resulted in 86% of accuracy of GEBVs from using SNPs on all 26 chromosomes (Daetwyler et al., 2012). As the relationship information is replicated on all chromosomes but the QTL effects are not, the majority of large SNP effects may be due to specific population structure and not due to QTLs.

Table 4 shows chromosomal positions and fraction of variances explained by the top 10 regions of the 4 methods: CGWAS, BayesB, ssGWAS1 and ssGWAS2. For ssGWAS1/3 and BayesB, the regions that accounted for the largest genetic variance were on chromosome 27 and identical, but accounted for vastly different amounts of genetic variance: 2.53 and 23.06%, respectively. The order of magnitude difference in genetic variance accounted for by the methods, even though the regions were the same, is due to how total genetic variance is accounted for by the methods. BayesB partitioned all the genetic variance to 10% of the SNPs while ssGWAS partitions the genetic variance among all SNPs. Thus, ssGWAS has more SNPs to distribute the same amount of genetic variance.

**Table 4:** **Rankings top 10 regions among different methods^a^**.

**CGWAS**	**chr^b^**	**gVar (%)^c^**	**ssGWAS1/3**	**gVar (%)**	**ssGWAS2/3**	**gVar (%)**	**BayesB**	**gVar (%)**
1^d^	6	3.07	2	1.29	62	0.38	2	2.35
2	6	2.9	3	0.91	110	0.26	3	1.89
3	6	1.3	4	0.78	8	0.84	40	0.25
4	6	0.98	360	0.09	810	0.01	322	0.06
5	6	0.79	278	0.11	565	0.02	27	0.32
6	27	0.79	1	2.53	1	5.65	1	23.06
7	6	0.6	668	0.04	1216	<0.01	1646	0
8	7	0.48	314	0.1	927	<0.01	99	0.14
9	12	0.48	855	0.03	925	<0.01	387	0.05
10	4	0.45	274	0.11	903	<0.01	173	0.09
Total^e^		11.84		5.99		7.16		28.21
**BayesB**	**chr**	**gVar (%)**	**ssGWAS1/3**	**gVar (%)**	**ssGWAS2/3**	**gVar (%)**	**CGWAS**	**gVar (%)**
1	27	23.06	1	2.53	1	5.65	6	0.79
2	6	2.35	2	1.29	62	0.38	1	3.07
3	6	1.89	3	0.91	110	0.26	2	2.9
4	11	1.39	15	0.43	31	0.55	279	0.08
5	2	1.03	42	0.28	63	0.38	656	0.04
6	3	1	144	0.16	166	0.18	11	0.43
7	4	0.73	9	0.53	105	0.27	450	0.06
8	5	0.68	6	0.59	16	0.72	423	0.06
9	2	0.59	7	0.56	57	0.39	32	0.29
10	2	0.54	264	0.11	119	0.24	53	0.22
Total		33.26		7.39		9.02		7.94
**ssGWAS1/3**	**chr**	**gVar (%)**	**ssGWAS2/3**	**gVar (%)**	**CGWAS**	**gVar (%)**	**BayesB**	**gVar (%)**
1	27	2.53	1	5.65	6	0.79	1	23.06
2	6	1.29	62	0.38	1	3.07	2	2.35
3	6	0.91	110	0.26	2	2.9	3	1.89
4	6	0.78	8	0.84	3	1.3	40	0.25
5	10	0.72	54	0.41	59	0.22	93	0.15
6	5	0.59	16	0.72	423	0.06	8	0.68
7	2	0.56	57	0.39	32	0.29	9	0.59
8	1	0.54	21	0.67	76	0.19	23	0.35
9	4	0.53	105	0.27	450	0.06	7	0.73
10	12	0.5	13	0.77	357	0.07	31	0.27
Total		8.95		10.36		8.95		30.32
c**ssGWAS2/3**	**chr**	**gVar (%)**	**ssGWAS1/3**	**gVar (%)**	**CGWAS**	**gVar (%)**	**BayesB**	**gVar (%)**
1	27	5.65	1	2.53	6	0.79	1	23.06
2	6	2.06	16	0.43	98	0.16	56	0.2
3	2	1.23	20	0.39	125	0.14	29	0.31
4	3	1.02	19	0.4	26	0.32	11	0.54
5	10	0.95	365	0.08	1063	0.02	77	0.17
6	2	0.92	370	0.08	573	0.05	155	0.1
7	14	0.85	82	0.21	606	0.05	41	0.25
8	6	0.84	4	0.78	3	1.3	40	0.25
9	2	0.83	13	0.45	123	0.14	14	0.41
10	12	0.83	152	0.15	555	0.05	118	0.13
Total		15.18		5.50		3.02		25.42

a *The third iteration of both scenarios (ssGWAS1/3 and ssGWAS2/3) in single-step genomic BLUP (ssGBLUP), BayesB, and classical genome wide association studies (CGWAS)*.

b *chr = chromosome number*.

c *gVar(%) = proportion of genetic variance each region consisting of 20 SNPs represents*.

d *Rankings of each region*.

e *Total = sum of gVar(%) of 10 regions of each method*.

Among the top 10 regions in ssGWAS1/3, there were 2, 4, and 6 regions respectively in common with ssGWAS2/3, CGWAS, and BayesB. In contrast, for the top 10 regions in BayesB, there were 6, 1, and 3 in common, respectively, with ssGWAS1/3, ssGWAS2/3 and CGWAS. Among the top 10 regions in CGWAS, there were 4, 3, 2, in common with ssGWAS1/ 3, BayesB, and ssGWAS2/3. Thus, in general, the rankings of top 10 regions were similar between ssGWAS (ssGWAS1 and ssGWAS2) and BayesB. The re-rankings in CGWAS was greater compared with the other methods. Additionally, the fraction of explained variance varied greatly among methods. Because of the way BayesB partitions variances among a fraction of the total SNPs, it is expected that BayesB will always assign a greater proportion of genetic variance to a SNP in any GWAS comparison.

The comparison between ssGBLUP and CGWAS is more direct. Part of the reason that CGWAS accounts for less genetic variance than ssGWAS may be because CGWAS does not take into account all relationships among subjects but only for genotyped individuals, which might lead to detection of spurious associations due to incompleteness (Kang et al., 2010). BayesB and CGWAS are also dependent on the choice of parameters and accuracy of deregression (Garrick et al., 2009; Van Hulzen et al., 2012), while ssGWAS1 or ssGWAS2 include all available relationships, and deregression is not necessary. Zeng et al. (2012) and Wang et al. (2012) examined a few methods for GWAS using simulated data sets, and both indicated that all methods were able to identify the same top few regions. However, few regions were common among methods in this study suggesting that simulations do not capture the complexities of real data and highlight the need to do comparisons using real data.

Many studies have looked at QTLs or chromosomal regions in chicken for body weight. For example, Rowe et al. (2006) looked for QTLs for 40-day body weight in Cobb-Vantress chickens. They found that chromosomal segments could explain up to 4% phenotypic variation (PV) on chromosomes 1, 4, and 5. Podisi et al. (2013) looked at body weight and gains at different ages for broiler-layer crosses. For body weight at 6 weeks, they identified several QTLs on chromosomes 1–4, 6, 8, 11, and 13 explaining >1.4% PV; the largest QTL was on chromosome 4 and explained 6.0% PV. Neither study found an important QTL on chromosome 27. The large proportion of explained variance could be due to simple models of analyses.

### Considerations

Windows were defined with fixed numbers of SNPs (i.e., 20), which might not match every pattern of haplotype blocks. Thus, over- or under-estimation of window variances were possible. Moreover, window variances were calculated based on SNP effects at each locus, which probably contains estimation errors, and translates into more variation in results for ssGWAS2. The noise due to the estimation process could be reduced by using sliding average values for SNP windows rather than point estimates.

Results showed that interpretation of GWAS using BayesB can be misleading. BayesB is based on a mixture model of those SNPs that explain genetic variance and those that do not (π). While the proportion of SNPs that explains genetic variance may become small, the total genetic variance remains constant, and is thus distributed among fewer SNPs resulting in what appears to be an inflated estimate of genetic variation accounted for by a SNP.

Every methodology for GWAS has a weakness. The ssGWAS1 method seems a more useful methodology compared with CGWAS and BayesB when a large number of phenotyped subjects are not genotyped, and obtaining deregressed proofs is difficult or impossible. A limitation of ssGWAS is that the number of iterations is dictated by heuristics at this time. Additional studies (unpublished) indicate that GWAS accuracy with ssGWAS1 is maximized at 2–4 iterations, with a single iteration creating noisy plots, and with more iterations suppressing signals from smaller QTLs. Another weakness of ssGWAS1 is the inability to determine the significance level. Possibilities to address this issue are the permutation test (Churchill and Doerge, 1994), or normalizing each SNP solution to a t-like statistic (McClure et al., 2012). The latter could be difficult to apply to a region including multiple SNPs. Future research may determine the level of significance in ssGWAS1 or ssGWAS2, e.g., following ideas by Garcia-Cortes and Sorensen (2001), where the estimation variances are obtained by sampling.

### Computing time

In this study, BayesB and CGWAS required DP which included running a regular BLUP, computing accuracies, and creating deregressed proofs. Omitting those procedures, GenSel required 17 h 13 min and WOMBAT required ~6 min. The very fast computing time in WOMBAT is due to precomputing matrices for prediction, so that computation for an additional marker takes very little time. Traditional algorithms were about 100 times slower for a population with about 1000 animals and 4000 SNP (Meyer and Tier, 2012). The ssGWAS methods were applied directly to the phenotypes without DP, and took about 15 min per iteration.

## Conclusion

This study compares genomic evaluation and association results between different methods: ssGWAS1, ssGWAS2, CGWAS, and BayesB. Because this was real data and the true values are unknown, it is not possible to conclude which method was most accurate for GWAS, but similarity between BayesB and ssGWAS1 was shown in various aspects. CGWAS was the most different but also found the greatest number of signals. The latter could be due to false positives. Advantages of using ssGWAS includes: (1) no pseudo values are required, (2) complex modeling and multiple-traits are possible, and (3) computing is fast and implementation is simple.

### Conflict of interest statement

The authors declare that the research was conducted in the absence of any commercial or financial relationships that could be construed as a potential conflict of interest.
